# A rigorous exploration of anal HPV genotypes using a next‐generation sequencing (NGS) approach in HIV‐infected men who have sex with men at risk for developing anal cancer

**DOI:** 10.1002/cam4.2720

**Published:** 2019-11-25

**Authors:** Chandrika J. Piyathilake, Suguna Badiga, Ranjit Kumar, Michael R. Crowley, Greer A. Burkholder, James L. Raper

**Affiliations:** ^1^ Department of Nutrition Sciences The University of Alabama at Birmingham (UAB) Birmingham AL USA; ^2^ UAB Center for Clinical & Translational Science The University of Alabama at Birmingham (UAB) Birmingham AL USA; ^3^ Department of Genetics The University of Alabama at Birmingham (UAB) Birmingham AL USA; ^4^ Division of Infectious Diseases Department of Medicine The University of Alabama at Birmingham (UAB) Birmingham AL USA

**Keywords:** anal cytology, HIV, HPV, MSM, next‐generation sequencing

## Abstract

**Background:**

There are no HPV‐based measures for managing anal cancer (AC) in HIV‐infected (HIV+) men who have sex with men (MSM) because of the high positivity of high‐risk (HR)‐HPVs. As next‐generation sequencing (NGS) is able to describe the composition of HPVs as percent (%) reads rather than positive vs negative results, we used NGS approach to detect HPVs in anal samples of HIV+ MSM to test its ability to differentiate those who are diagnosed with atypical squamous cells of unknown significance or greater (ASCUS+) from those who are free of such lesions and to understand the burden of HPV infections in relation to HPV vaccines.

**Methods:**

Study included 81 HIV+ MSM characterized for demographics, patient‐reported outcome measures, HIV related laboratory measures and anal cytology. We summarized NGS HPV data using % read cut points (>0%‐>30%) and tested the relationship between % reads of HR‐HPVs and risk of ASCUS+ using logistic regression.

**Results:**

Forty‐six HPVs were detected at the >0% read cut point. The prevalence of any HR‐HPVs varied from 100% to 40% with >0% to >30% reads while ≥99% were infected with HR‐HPVs included or not included in the 9 valent HPV vaccine at the >0% read cut point. MSM with >30% HR‐HPV reads were 4.5 times more likely to be diagnosed with ASCUS+ compared to ≤30% reads (*P *= .033).

**Conclusion:**

NGS‐based approach is more accurate than PCR‐based HPV testing for identifying HIV+ MSM at risk for developing AC. We raise the concern regarding the efficacy of current HPV vaccines for preventing AC in this high‐risk population.

## Graphical abstract

Next‐generation sequencing‐based approach is more accurate than PCR‐based HPV testing for identifying HIV+ MSM at risk for developing anal cancer. We raise the concern regarding the efficacy of current HPV vaccines for preventing anal cancer in this high‐risk population.

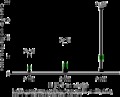

## 1 BACKGROUND

Therapy with combination antiretroviral treatment (cART) has reduced the risk of acquired immune deficiency syndrome (AIDS) and dramatically prolonged the survival of people living with HIV (PLWH) in developed countries.^1^ A consequence of this trend has been an increase in non‐AIDS‐defining cancers (NADCs) including cancers of the anus^2^ in this population. A substantial proportion of the increase in incident NADCs is attributed to cancers caused by infections with carcinogenic or high‐risk (HR)‐human papillomaviruses (HPVs). Of these, the largest increase was seen for anal intraepithelial neoplasia (AIN) and anal cancer (AC). Compared with the general population,^3^ PLWH are 19 times more likely to be diagnosed with AC.^4^ More importantly, a marked increase in the incidence of AC had occurred after the introduction of cART, indicating that immune restoration with cART is unlikely to prevent the increased risk of HPV associated AC.^5^ With a incidence rate of 77‐137 per 100 000, exceeding the rate of cervical cancer in countries without an organized screening program, HIV‐infected (HIV+) men who have sex with men (MSM) have the highest risk of developing AC.^6^

According to the CDC,^7^ data are insufficient to recommend routine AC screening with anal cytology in PLWH, MSM without HIV infection, and the general population. The CDC suggests that an annual digital anorectal examination may be useful to detect masses on palpation that could be AC in PLWH, especially in those with a history of receptive anal intercourse. Some clinical centers perform anal cytology to screen for AC among high‐risk populations (eg, HIV+ MSM with a history of receptive anal intercourse), followed by high‐resolution anoscopy (HRA) for those with abnormal cytologic results (eg, ASCUS+). Lastly, even though there is little doubt that the excess risk of AC in HIV+ MSM is largely due to the higher prevalence of HPV infections, the CDC states that routine oncogenic HPV tests are not considered clinically useful for AC screening among MSM because of the high prevalence of anal HPV infections. Therefore, currently there are no established HPV‐based screening guidelines, triage or preventive measures for controlling AC in HIV+ MSM. Despite that studies report that in HIV+ MSM, HPV infections could be nearly universal^8^, ^9^ and therefore HPV testing may not yield adequate predictive value for triage of patients for further care.^10^ Whether this HPV prevalence data is based on any HPV or HR‐HPV, however, is somewhat unclear. A systematic review and meta‐analysis that mainly included studies in Europe and North America and detected HPVs using broad‐spectrum polymerase chain reaction (PCR)‐based assays reported 81%, 30% and ~10%‐15% prevalence of any anal HPV, HPV 16 and non‐HPV16 HR‐HPV genotypes, respectively among HIV+ MSM irrespective of anal cytological diagnoses.^11^ A similar or slightly higher any HPV prevalence was reported for HIV+ MSM in China,^12^ Mexico,^13^, ^14^ Spain^15^, ^16^ and Italy.^17^ In‐depth HPV genotyping based on next‐generation sequencing (NGS) rather than kit‐based assays are likely more capable of separating high risk AC individuals from low risk AC individuals because NGS is able to describe the composition of specific HPV genotypes as % reads for each genotype in a given specimen rather than positive vs negative results provided by the kit‐based PCR assays. However, there is a lack of studies using NGS among HIV+ MSM, indicating the need for studies of this nature.

Even though vaccines may potentially prevent high‐risk anal HPV infections, currently available vaccination protocols and clinical guidelines would certainly benefit from in‐depth HPV genotyping of anal samples. In order to address these gaps in knowledge, we used a NGS approach to explore HPV genotypes present in anal samples of HIV+ MSM with any of the following cytologic findings: 1) negative for intraepithelial lesions or malignancy (NILM), 2) atypical squamous cells of unknown significance (ASCUS), 3) low‐grade squamous intraepithelial lesions (LSIL) and 4) high‐grade squamous intraepithelial lesions (HSIL).

## 2 METHODS

### 2.1 Study population

Study included 81 HIV+ MSM with a history of anal receptive intercourse who were seen at the UAB's 1917 HIV Outpatient Clinic in Birmingham, AL. The clinic provides comprehensive core medical and social services to adult PLWH. All patients were characterized for demographics, patient‐reported outcome measures (PROs),^18^ HIV‐related laboratory measures and anal cytology as routine care. All patients were on cART at time of study sample collection with a median duration on cART for 5.0 years and an inter quartile range of 1.5‐9.0 years. For the purposes of this study, data from the clinic visit completed closest to the sample collection visit (eg age) as well as multiple data points available over a period were used (eg nadir CD4 and CD4 at the time of anal cytology/HPV testing). Main variables of interest for the current study were age, race, body mass index (BMI), smoking, life‐time number of partners, CD4 count and quantitative HIV viral RNA load.

A medical provider (physician or nurse practitioner) collected anal samples using clinic standard collection protocols. Briefly, cells were collected using a water‐moistened, synthetic‐fiber swab with a non‐scored stick. A swab was inserted into the anal canal past the dentate line until it abuts the distal rectal wall. Cells were harvested by using a circular motion as the swab was retracted while applying firm lateral pressure to sample the epithelium in the mucosal folds of the anal canal. Swabs were rinsed in Thin Prep Pap Test^®^ solution by agitating the swab in the solution 10 times and pushing it against the PreservCyt vial wall to further release cells. Vial caps were tightened so the torque line on the cap passes the torque line on the vial. These vials were labeled at the bedside and transported to the UAB cytopathology laboratory for cytological testing. Immediately after the cytology tests were completed, all samples were delivered to the laboratory of Dr Piyathilake for processing and storage for HPV NGS assay. The anal cytological diagnoses were obtained from the UAB patient database (NILM, n = 24; ASCUS, n = 27; LSIL, n = 25; HSIL, n = 5). The study protocol and procedures were approved by the UAB Institutional Review Board.

### 2.2 Laboratory assay protocols

#### 2.2.1 DNA extractions

DNA was isolated using the fecal DNA isolation kit from Zymo Research following the manufacturer's instructions. DNA concentrations/quality was determined based on 260/280 ratio using a nanodrop 2000 spectrophotometer. DNA with a 260/280 ratio of 1.7‐2.0 was considered satisfactory.

#### 2.2.2 NGS of HPVS

To ensure the quality of results generated from the proposed study, the HPV sequencing assay described below included two positive control DNA specimens (Hela Cell DNA/HPV 18 and Caski/HPV 16) in each PCR run. Sequencing results of >98% reads for each HPV genotype was required to accept the sequencing results for patient specimens in a given PCR run.

Analysis of various HPV sequences was accomplished with deep sequencing of 20 ng of DNA isolated from each anal sample. Methods followed those described previously in the human papillomavirus laboratory manual from the WHO^19^ for HPV amplification from patient material with modifications. Briefly, three successive rounds of PCR were performed to isolate HPV specific sequences. The first round of PCR with a mixture of primers for PGMY11/09 and the second round with nested HPV PCR primers GP5+/GP6+ (GP5+ 5′ CTACACGACGCTCTTCCGATC‐TTTTGTTACTGTDGTDGAYACYAC 3′ and GP6+ 5′ GTTCAGACG‐TGTGCTCTTCCGATCGA‐AAHAY‐AAAYTGYAADTCAWAYTC 3′) that also contained sequences at their 5′ termini to incorporate sequences necessary for cluster formation on the MiSeq flowcells and to include sequences for a dual indexing scheme. The addition of Illumina TruSeq specific sequence was done by PCR in Herculase enzyme mix with initial denaturation at 98°C for 2 minutes followed by a two‐step protocol consisting of 4 cycles of 98°C 20 seconds, 55°C for 20 seconds, 72°C for 20 seconds then an additional 8 cycles at 98°C for 20 seconds, 62°C for 20 seconds, 72°C for 20 seconds. The final target sequence was approximately 240 bp in length; therefore, we ran paired end 250 bp sequencing reactions on the MiSeq (Illumina). Following sequencing on the MiSeq, the raw sequence reads were converted to fastq files using Illumina software then aligned to the HPV genome to determine variants within each sample. Sequence quality of HPV sequences was measured using tool FASTQC and low quality reads (average Q < 20) were filtered using FASTX. An HPV sequence BLAST database was constructed from all Reference genomes for HPV available from https://pave.niaid.nih.gov (PaVE database). Megablast was used to search each read against the database and top hits with specified given identity cutoff of 90% match and coverage <70% were identified. The proportion of hits for a given sample described the composition of specific HPV genotypes as % reads for each genotype in a given sample that adds to 100% with a certain percentage of sequences that did not match with sequences in the PAVE database. Figure S1 shows NGS HPV genotype results for five anal specimens to demonstrate the distribution of % reads of HPV genotypes in a given specimen. For example, in specimen 1, 75.686% of reads belong to HPV genotype 35 (49528/65282 × 100 = 75.686%).

### 2.3 Data analysis

The differences in the characteristics of MSM diagnosed with NILM or ASCUS+ were tested using Pearson chi square test. The prevalence of HPV genotypes was summarized using the following % read cut points; >0%,>5%, >10%,>20% and >30%. Because of the universal presence of HR‐HPVs at ≤5% reads, to test the association between HR‐HPV genotypes and ASCUS+, we created the HR‐HPV variable by averaging the % reads of all HR‐HPVs with >5% reads in a sample. Afterward, the relationship between % reads of HR‐HPVs (cut points >10%,>20% and >30% reads) and the risk of being diagnosed with ASCUS+ was tested using unconditional logistic regression models after adjusting for age, race, body mass index (BMI), smoking status, lifetime number of sexual partners and improvement in CD4 (calculated based on the difference between CD4 count at time of diagnosis/HPV testing and nadir CD4 count) or the CD4 count and viral load at time of diagnosis/HPV testing.

## 3 RESULTS

Table 1 shows the differences in the demographics, other relevant variables, indicators of HIV status and HR‐HPV % read cut point between MSM diagnosed with NILM and those diagnosed with ASCUS+. Improvement in CD4 was ≤500 in a large percentage of MSM diagnosed with ASCUS+ (86%) compared to those diagnosed with NILM (64%) (*P *= .0269). Approaching significance (*P *= .0731), a higher percentage of MSM (82%) diagnosed with NILM had undetectable HIV viral loads (<20 copies/mL) compared to MSM diagnosed with ASCUS+ (61%). No other variables were significantly different between MSM diagnosed with NILM and those diagnosed with ASCUS+.

**Table 1:** Differences in demographic and other relevant variables by anal cytological diagnosis

Variables	ASCUS+*, *	NILM**, **	*P*
Age (years)			
<32	17 (30%)	10 (42%)	.302
≥32	40 (70%)	14 (58%)	
Race			
African American	31 (54%)	16 (67%)	.307
Caucasian American	26 (46%)	8 (33%)	
BMI (kg/m^2^)			
<25	22 (47%)	9 (41%)	.646
≥25	25 (53%)	13 (59%)	
Smoking status			
Current	27 (47%)	10 (42%)	.638
Non‐current	30 (53%)	14 (58%)	
Lifetime number of partners			
<30	10 (18%)	8 (33%)	.119
≥30/undisclosed	47 (82%)	16 (67%)	
Improvement in CD4 (cells/mm^3^)			
>500	8 (14%)	8 (36%)	.027
≤500	49 (86%)	14 (64%)	
Current CD4 count (cells/mm^3^) at the time of diagnosis/HPV testing			
>500	35 (61%)	16 (73%)	.346
≤500	22 (39%)	6 (27%)	
Viral loads (copies/mL) at the time of diagnosis/HPV testing			
Detectable (≥20)	22 (39%)	4 (18%)	.073
Undetectable (<20)	35 (61%)	18 (82%)	
HR‐HPV % reads			
>30%	25 (44%)	7 (29%)	.217
≤30%	32 (56%)	17 (71%)	

Note

* ASCUS+, higher than atypical squamous cell of undetermined significance.

** NILM, negative for intraepithelial lesions or malignancy.

**Figure 1:**
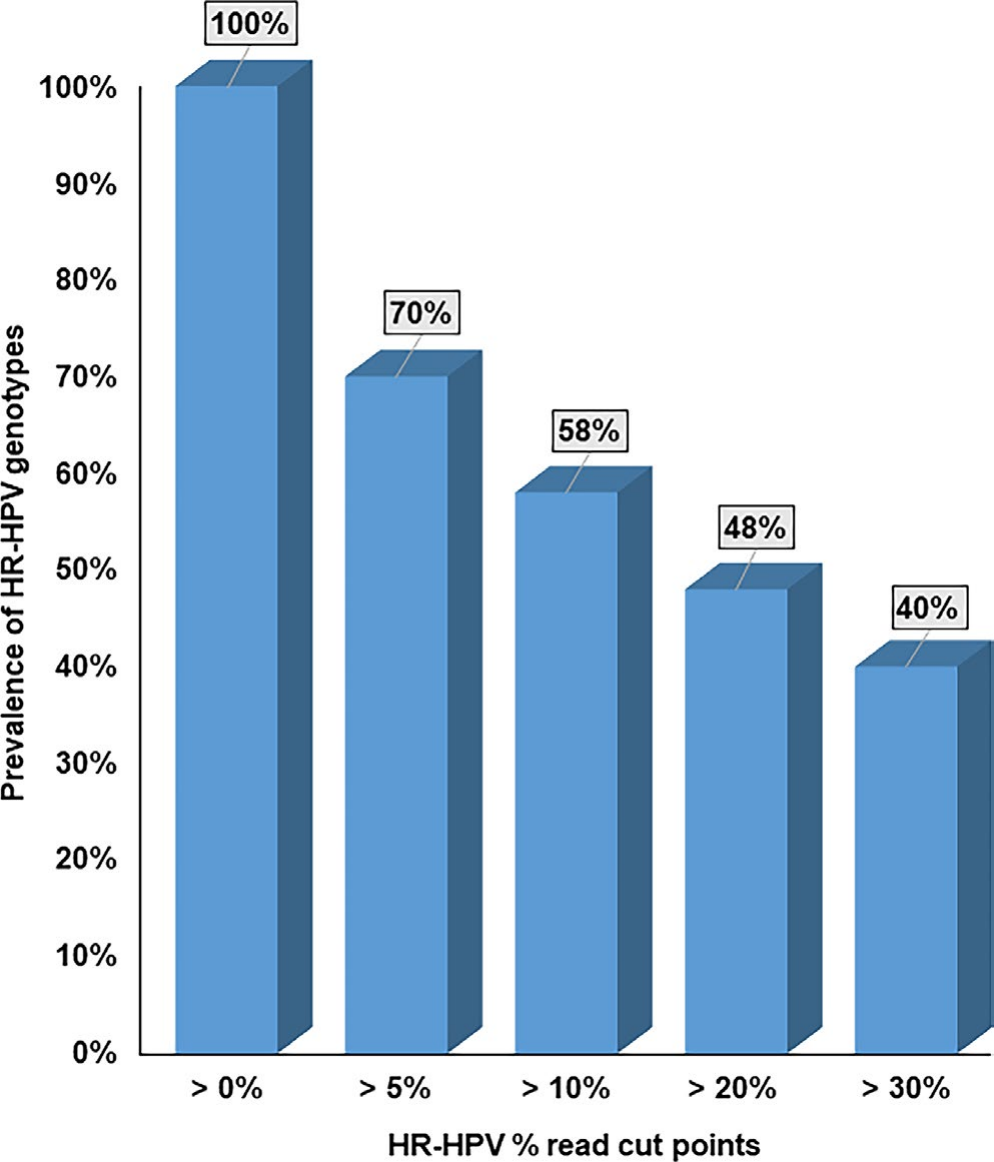
The prevalence of HR‐HPV genotypes by % read cut points

**Figure 2:**
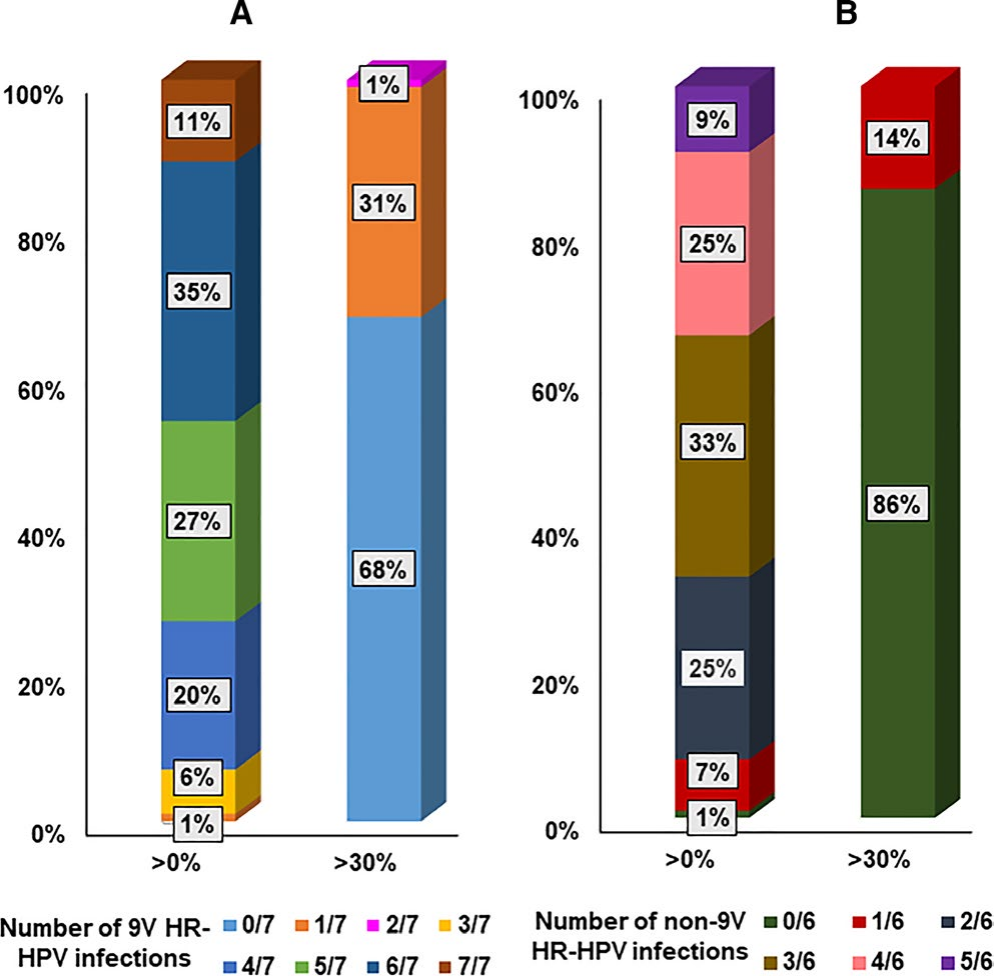
The percentage of the population with single or multiple infections of A) 9V vaccine HR‐HPV genotypes and B) non‐9V vaccine HR‐HPV genotypes based on % read cut points

**Figure 3:**
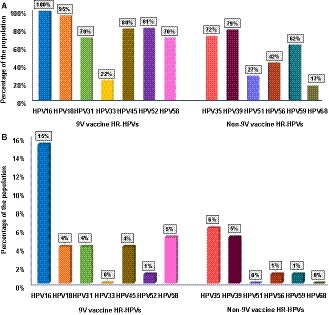
The prevalence of 9V vaccine HR‐HPV genotypes and non‐9V vaccine HR‐HPV genotypes with % reads of A) >0%, B) >30%

As shown in the Table S1, a total of 46 HPV genotypes that included 13 known HR‐HPVs, 21 known low‐risk (LR) HPVs, 12 HPVs documented in the PaVE database and a collection of HPV sequences currently not documented in the PaVE database were detected at the >0% read cut point. The prevalence rates of all HPVs decreased from >0‐>30% read cut points. The prevalence of known HR‐HPV genotypes in HIV+ MSM varied from 100% (universal) to 40% with the lowest % read cut point (>0) to the highest cut point (>30), respectively (Figure 1). 100% and 99% of HIV+ MSM were infected with at least one HR‐HPV included in the 9V HPV vaccine or HR‐HPVs not included in 9V HPV vaccine respectively at the >0% read cut point. More than 90% of HIV+ MSM were infected with 4‐7 HR‐HPV genotypes included in the 9V HPV vaccine or 2‐6 HR‐HPVs not included in the 9V HPV vaccine at the > 0% read cut point. The prevelance of those multiple infections decreased at higher cutpoints (Figure 2A,B). Further, we also observed a similar prevalence of certain HR‐HPV genotypes not included in the 9V HPV vaccine compared to HR‐HPV genotypes included in the 9V HPV vaccine at all % read cut points, including >0% cut point (Figure 3A) as well as at the highest cut point (>30%), Figure 3B.

**Figure 4:**
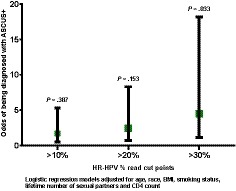
The odds of being diagnosed with any anal abnormal cytology (atypical squamous cells of undetermined significance [ASCUS]+) by HR‐HPV % read cut points

There was a stepwise increase in the odds of being diagnosed with any abnormal anal cytology from >10% reads (OR = 1.70) to >30% reads of HR‐HPVs (OR = 4.53) (Figure 4), independent of covariates. The full regression model depicting the relationship between HR‐HPVs based on the >30% read cut point and the risk of being diagnosed with ASCUS+ is presented in Table 2. HIV+ MSM with >30% HR‐HPV reads were 4.5 times more likely to be diagnosed with ASCUS+ compared to ≤30% reads (*P *= .033). MSM ≥ 32 years of age were 4.3 times more likely to be diagnosed with ASCUS+ compared to < 32 years (*P* = .041). MSM who did not achieve an improvement in CD4 levels on cART >500 were 8.3 times more likely to be diagnosed with ASCUS+ compared to those who improved >500 (*P* = .006). The association between HR‐HPV and risk of being diagnosed with ASCUS+ remained similar when we replaced the improvement in CD4 with either CD4 or viral load at the time of diagnosis/HPV testing (OR = 4.09, 95%CI = 1.10‐15.30, *P *= .036 and OR = 4.87, 95%CI = 1.23‐19.30, *P *= .024), respectively. However, we did not observe a statistically significant association between CD4 or viral load at the time of diagnosis/HPV testing and risk of being diagnosed with ASCUS+ (OR = 2.29, 95%CI = 0.67‐7.88, *P *= .188 and OR = 3.9, 95%CI = 0.93‐16.70, *P *= .063, respectively).

**Table 2:** Regression model depicting the association between HR‐HPV % reads cut point >30% and risk of being diagnosed with ASCUS+

Independent variables	ASCUS+*, * vs NILM**, **	
	OR (95%CI)	*P*
Age (years)		
<32	1.00	.041
≥32	4.30 (1.05‐17.80)	
Race		
African American	1.00	.674
Caucasian American	1.33 (0.35‐4.96)	
BMI (kg/m^2^)		
<25	1.00	.370
≥25	0.57 (0.17‐1.92)	
Smoking status		
Non‐current	1.00	.727
Current	1.25 (0.36‐4.28)	
Life time number of partners		
<30	1.00	.054
≥30/undisclosed	4.49 (0.98‐20.60)	
Improvement in CD4 counts (cells/mm^3^)		
>500	1.00	.006
≤500	8.30 (1.82‐38.80)	
HR‐HPV (% reads)		
≤30	1.00	.033
>30	4.53 (1.13‐18.10)	

* ASCUS+, higher than atypical squamous cell of undetermined significance.

** NILM, negative for intraepithelial lesion or malignancy.

## 4 DISCUSSION

Previous studies that compared NGS with other HPV detection methods such as INNO‐LiPA DNA hybridization, Electrochemical DNA Chip, Roche Linear Array HPV genotyping (LA) and multiplex PCR reported that NGS is more sensitive and has the ability to detect multiple infections compared to traditional methods and, therefore, may have the potential for use as an alternative method for HPV genotyping and diagnosis of lesions.^20^, ^21^, ^22^, ^23^, ^24^, ^25^ However, to our knowledge, our study is the first to investigate the presence of anal HPV genotypes using an NGS‐based approach and report their prevalence as varying % read cut points in HIV+ MSM characterized for anal lesion status, demographic and other relevant risk factors.

The most prevalent HR‐HPV genotype detected in our study was HPV 16 at all cut points, including the highest cut point, >30% cut point which was significantly associated with the risk of ASCUS+. Other HPV types with higher prevalence in general were HPV 18 followed by HPV 52, 35, 39 and 45. In contrast, a study that detected HPVs using 454 NGS and reported results based on the total number of HPV reads in anal specimens of HIV+ MSM in Mexico, documented that HPV 45 was the most prevalent followed by HPV 51, 58, 16, 52 and 59.^26^ That study and ours identified most of the known LR‐HPVs and the following HPV genotypes that are not classified as HR or LR; HPV 44, 32, 74, 85, 86, 102 and 97. HPV 44 and 74 have been previously detected in cervical specimens of HIV+ women.^27^, ^28^ Additionally, we identified HPV 114 in our study which was previously reported in the cervix of HIV+ women.^29^

Our observation that the prevalence of known HR‐HPVs in HIV+ MSM varies from 100% (universal) to 40% with the lowest % read cut point to the highest cut point and there was a stepwise increase in the odds of being diagnosed with any abnormal anal cytology from low to high % reads indicated that NGS‐based HPV testing is likely to be more accurate than PCR‐based HPV testing to identify HIV+ MSM at risk for lesions that may progress to AC. This would allow for targeted referral of HIV+ MSM for high‐resolution anoscopy (HRA) in a cost effective way. A previous study conducted among HIV negative (HIV‐) MSM documented that having > 5 anal sex partners and PCR evidence of an anal HPV infection but not age or smoking status were significantly associated with any abnormal anal cytology diagnoses.^30^ In our study, older men were approximately four times more likely to be diagnosed with ASCUS+. Age related differences in sexual practices in HIV+ MSM may explain the differences in these results between HIV+ and HIV‐ men. We observed that HIV+ MSM with HR‐HPV reads >30% were significantly more likely to be diagnosed with ASCUS+ compared to those with ≤30% reads. A plausible link between higher % reads of HR‐HPVs and HPV integration, a key event in the HPV carcinogenic process may explain this association as integrated HPV genome copies are clearly associated with advanced HPV related precancerous lesions.^31^, ^32^ Interestingly, our results also revealed that a lower improvement in CD4 from nadir CD4 rather than lower CD4 or viral load at the time of lesion diagnosis are likely to be better predictors of AC risk thereby suggesting the importance of adherence to cART to improve and maintain CD4 counts.^33^ A previous study reported that among HIV+ men, those with lower baseline CD4 counts were more likely to develop anal HSIL.^34^

With regard to the primary prevention of AC, HPV vaccination has been recommended in some countries for MSM under 45 years of age^35^ or under the age of 27 years.^36^ Our NGS‐based HPV genotyping showed that >90% of HIV+ MSM are infected with 4‐7 HR‐HPV genotypes included in the 9V HPV vaccine at the >0% read cut point and the prevelance of multiple infections decreased at higher cut points. It is unclear whether vaccine efficacy would be affected by having lower % reads of those HPVs. Future studies that test the relationship between a range of % reads and their relationship to incident high grade anal intraepithelail neoplasia (AIN 2+) in a vaccinated population are needed to address this concern. Our observation of a similar prevalence of certain HR‐HPV genotypes not included in the 9V HPV vaccine compared to HR‐HPV genotypes included in the 9V HPV vaccine at all % read cut points, including the highest cut point, raises the concern of 9V HPV vaccine efficacy to prevent AC in this population. We previously demonstrated that higher grades of cervical intraepithelial neoplasia (CIN 2+) lesions that develop due to HR‐HPV genotypes not included in 9V HPV vaccine have similar malignant potential to that of CIN 2+ due to HR‐HPV genotypes included in the 9V HPV vaccine^37^ indicating the importance of evaluating the effects of those HPV genotypes on incident AIN 2+ in future studies.

Our study has several strengths including a population representative of the HIV+ MSM receiving care in the Deep South, which is disproportionately impacted by the HIV epidemic in the United States; a good assessment of anal cytology/risk factors and state of the art HPV testing. Even though NGS data may be relatively complex to analyze, it may serve as a cost effective and sensitive HPV genotyping method because of its highly sensitive detection capability of multiple HPV genotypes and the ability to associate HPV risk based on HPV sequence % reads, allowing for a accurate measure rather than a less accurate nominal “positive vs negative” measure for a given HPV genotype. However, discovery of simple biomarkers of HPV carcinogenesis that are strongly related to NGS HPV results are needed to make use of this approach effectively in clinical settings. Our results will form the foundation for discovery and validation of such biomarkers.

Limitations of the study are the cross‐sectional study design which did not allow us to examine the temporal association between exposure to varying % reads of HPV sequences and risk of developing ASCUS+ and lack of histological diagnoses of lesions since only a few patients were referred for HRA directed biopsy in accordance with the current 1917 Clinic protocol. Since the ASCUS and LSIL cytology diagnoses are likely to be upgraded to higher grades on biopsy,^38^, ^39^, ^40^, ^41^ there is likelihood of misclassification of patients by diagnosis. However, any potential under or over diagnosis of abnormal cytology probably does not affect the estimates of associations with potential risk factors of interest since we categorized lesion diagnoses in all models as NILM vs. ASCUS+.

In conclusion, we report that our NGS‐based approach is more accurate than PCR‐based HPV testing to identify HIV+ MSM at risk for developing AC as MSM with >30% HR‐HPV reads were 4.5 times more likely to be diagnosed with ASCUS+ compared to ≤30% reads. We also raise the concern regarding the efficacy of currently available HPV vaccines for preventing AC in this high risk population as HIV+ MSM are universally exposed to HR‐HPV genotypes that are included or not included in such vaccines. However, our results should not be interpreted in a way to discourage vaccination of HIV+ with currently available HPV vaccines. Replication of all our findings in other HIV populations is needed to increase the scientific credibility of these observations.

## AUTHOR'S CONTRIBUTIONS

Formulation of research goals and aims‐CJP. Generation of data and verification‐MRC, SB, CJP, GAB, JLR. Data analysis and interpretation‐RK, SB, CJP. Writing original draft and presentation of results‐CJP, SB. Review and editing of the final manuscript‐CJP, SB, RK, MRC, GAB, JLR.

## Data Availability

Data is available for sharing.

## References

[1] Samji H , Cescon A , Hogg RS , et al. American AIDS Cohort Collaboration on Research and Design (NA‐ACCORD) of IeDEA . Closing the gap: increases in life expectancy among treated HIV‐positive individuals in the United States and Canada. PLoS One. 2013;8:e81355. 24367482 10.1371/journal.pone.0081355 PMC3867319

[2] Deeken JF , Tjen‐A‐Looi A , Rudek MA , et al. The rising challenge of non‐AIDS‐defining cancers in HIV‐infected patients. Clin Infect Dis. 2012;55:1228‐1235. 22776851 10.1093/cid/cis613 PMC3529613

[3] de Martel C , Plummer M , Vignat J , Franceschi S . Worldwide burden of cancer attributable to HPV by site, country and HPV type. Int J Cancer. 2017;141:664‐670. 28369882 10.1002/ijc.30716 PMC5520228

[4] Hernández‐Ramírez RU , Shiels MS , Dubrow R , Engels EA . Cancer risk in HIV‐infected people in the USA from 1996 to 2012: a population‐based, registry‐linkage study. Lancet HIV. 2017;4:e495‐e504. 28803888 10.1016/S2352-3018(17)30125-X PMC5669995

[5] Piketty C , Selinger‐Leneman H , Grabar S , et al. Marked increase in the incidence of invasive anal cancer among HIV‐infected patients despite treatment with combination antiretroviral therapy. AIDS. 2008;22:1203‐1211. 18525266 10.1097/QAD.0b013e3283023f78

[6] Ferlay J , Soerjomataram I , Ervik M , et al. eds. Globocan 2012: estimated cancer incidence, mortality, and prevalence worldwide in 2012. IARC CancerBase No. 11. Lyon, France: International Agency for Research on Cancer (IARC); 2018 Available at http://globocan.iarc.fr/Default.aspx. Accessed January 15, 2018.

[7] Centers for Disease Control and Prevention (CDC) . Sexually transmitted diseases treatment guidelines. MMWR Recommendations and Reports, 64(RR‐03); 2015 https://www.cdc.gov/std/tg2015/hpv-cancer.htm. Accessed August 13, 2019.

[8] Critchlow CW , Holmes KK , Wood R , et al. Association of human immunodeficiency virus and anal human papillomavirus infection among homosexual men. Arch Intern Med. 1992;152:1673‐1676. 1323247

[9] Palefsky JM , Holly EA , Ralston ML , Jay N . Prevalence and risk factors for human papillomavirus infection of the anal canal in human immunodeficiency virus (HIV)‐positive and HIV‐negative homosexual men. J Infect Dis. 1998;177:361‐367. 9466522 10.1086/514194

[10] Viciana P , Milanés‐Guisado Y , Fontillón M , et al. High‐risk human papilloma virus testing improves diagnostic performance to predict moderate‐to‐high grade anal intraepithelial neoplasia in HIV‐infected men who have sex with men in low‐to‐absent cytological abnormalities. Clin Infect Dis. 2019; pii:ciz144. 10.1093/cid/ciz144 30770528

[11] Marra E , Lin C , Clifford GM . Type‐specific anal human papillomavirus prevalence among men, according to sexual preference and HIV status: a systematic literature review and meta‐analysis. J Infect Dis. 2019;219:590‐598. 30239749 10.1093/infdis/jiy556

[12] Li X , Li M , Yang YU , et al. Anal HPV/HIV co‐infection among Men who have sex with men: a cross‐sectional survey from three cities in China. Sci Rep. 2016;6:21368. 26892938 10.1038/srep21368 PMC4759533

[13] Méndez‐Martínez R , Rivera‐Martínez NE , Crabtree‐Ramírez B , et al. Multiple human papillomavirus infections are highly prevalent in the anal canal of human immunodeficiency virus‐positive men who have sex with men. BMC Infect Dis. 2014;14:671. 25510243 10.1186/s12879-014-0671-4 PMC4272559

[14] Torres‐Ibarra L , Conde‐Glez CJ , Salmerón J , et al. Risk factors for anal HPV‐16/18 infection in Mexican HIV‐infected men who have sex with men. Prev Med. 2014;69:157‐164. 25251099 10.1016/j.ypmed.2014.09.011

[15] del Amo J , González C , Geskus RB , et al. What drives the number of high‐risk human papillomavirus types in the anal canal in HIV‐positive men who have sex with men? J Infect Dis. 2013;207:1235‐1241. 23325914 10.1093/infdis/jit028

[16] Torres M , González C , del Romero J , et al. Anal human papillomavirus genotype distribution in HIV‐infected men who have sex with men by geographical origin, age, and cytological status in a Spanish cohort. J Clin Microbiol. 2013;51:3512‐3520. 23966501 10.1128/JCM.01405-13 PMC3889727

[17] Latini A , Gabriella Dona M , Ronchetti L , et al. Prevalence of anal human papillomavirus infection and cytologic abnormalities among HIV‐infected and HIV‐uninfected men who have sex with men. J Int AIDS Soc. 2014;17:19662. 25397412 10.7448/IAS.17.4.19662 PMC4225312

[18] Kozak MS , Mugavero MJ , Ye J , et al. Patient reported outcomes in routine care: advancing data capture for HIV cohort research. Clin Infect Dis. 2012;54:141‐147. 22042879 10.1093/cid/cir727 PMC3243652

[19] Human papillomavirus laboratory manual. First edition; 2009 Geneva, Switzerland: WHO Press, World Health Organization. (WHO/IVB/10.12).

[20] da Fonseca AJ , Galvão RS , Miranda AE , Ferreira LC , Chen Z . Comparison of three human papillomavirus DNA detection methods: next generation sequencing, multiplex‐PCR and nested‐PCR followed by sanger based sequencing. J Med Virol. 2016;88:888‐894. 26496186 10.1002/jmv.24413

[21] Nilyanimit P , Chansaenroj J , Poomipak W , Praianantathavorn K , Payungporn S , Poovorawan Y . Comparison of four human papillomavirus genotyping methods: next‐generation sequencing, INNO‐LiPA, electrochemical DNA Chip, and Nested‐PCR *Ann* . Lab Med. 2018;38:139‐146. 10.3343/alm.2018.38.2.139 PMC5736673 29214758

[22] Escobar‐Escamilla N , Ramírez‐González JE , Castro‐Escarpulli G , Díaz‐Quiñonez JA . Utility of high‐throughput DNA sequencing in the study of the human papillomaviruses. Virus Genes. 2018;54:17‐24. 29282656 10.1007/s11262-017-1530-3

[23] Arroyo LS , Smelov V , Bzhalava D , Eklund C , Hultin E , Dillner J . Next generation sequencing for human papillomavirus genotyping. J Clin Virol. 2013;58:437‐442. 23932809 10.1016/j.jcv.2013.07.013

[24] Nowak RG , Ambulos NP , Schumaker LM , et al. Genotyping of high‐risk anal human papillomavirus (HPV): ion torrent‐next generation sequencing vs. linear array. Virol J. 2017;14:112. 28610586 10.1186/s12985-017-0771-z PMC5470268

[25] Meiring TL , Salimo AT , Coetzee B , et al. Next‐generation sequencing of cervical DNA detects human papillomavirus types not detected by commercial kits. Virol J. 2012;9:164. 22897914 10.1186/1743-422X-9-164 PMC3493284

[26] González‐Hernández LA , Flores‐Miramontes MG , Aguilar‐Lemarroy A , et al. HPV genotypes detected by linear array and next‐generation sequencing in anal samples from HIV positive men who have sex with men in Mexico. Arch Virol. 2018;163:925‐935. 29299683 10.1007/s00705-017-3697-2

[27] Longuet M , Cassonnet P , Orth G . A novel genital human papillomavirus (HPV), HPV type 74, found in immunosuppressed patients. J Clin Microbiol. 1996;34:1859‐1862. 8784613 10.1128/jcm.34.7.1859-1862.1996 PMC229138

[28] Belglaiaa E , Elannaz H , Mouaouya B , et al. Human papillomavirus genotypes among women with or without HIV infection: an epidemiological study of Moroccan women from the Souss area. Infect Agent Cancer. 2015;10:44. 26664495 10.1186/s13027-015-0040-y PMC4673842

[29] Badial RM , Dias MC , Stuqui B , et al. Detection and genotyping of human papillomavirus (HPV) in HIV‐infected women and its relationship with HPV/HIV co‐infection. Medicine (Baltimore). 2018;97:e9545. 29620669 10.1097/MD.0000000000009545 PMC5902291

[30] Chin‐Hong PV , Vittinghoff E , Cranston RD , et al. Age‐related prevalence of anal cancer precursors in homosexual men: the EXPLORE study. J Natl Cancer Inst. 2005;97:896‐905. 15956651 10.1093/jnci/dji163

[31] Klaes R , Woerner SM , Ridder R , et al. Detection of high‐riskcervical intraepithelial neoplasia and cervical cancer by amplifi‐cation of transcripts derived from integrated papillomavirusoncogenes. Cancer Res. 1999;59:6132‐6136. 10626803

[32] Luft F , Klaes R , Nees M , et al. Detection of integratedpapillomavirus sequences by ligation‐mediated PCR (DIPS‐PCR)and molecular characterization in cervical cancer cells. IntJ Cancer. 2001;92:9‐17. 11279600

[33] Nachega JB , Marconi VC , van Zyl GU , et al. HIV treatment adherence, drug resistance, virologic failure: evolving concepts. Infect Disord Drug Targets. 2011;11:167‐174. 21406048 10.2174/187152611795589663 PMC5072419

[34] Palefsky JM , Holly EA , Ralston ML , Jay N , Berry JM , Darragh TM . High incidence of anal high‐grade squamous intra‐epithelial lesions among HIV‐positive and HIV‐negative homosexual and bisexual men. AIDS. 1998;12:495‐503. 9543448 10.1097/00002030-199805000-00011

[35] Joint Committee on Vaccination and Immunisation . JCVI interim position statement on HPV vaccination of men who have sex with men. 2017 https://assets.publishing.service.gov.uk/government/uploads/system/uploads/attachment_data/file/373531/JCVI_interim_statement_HPV_vacc.pdf. Accessed July 7, 2018.

[36] Government of Canada . Canadian immunization guide: Part 4‐Active vaccines.https://www.canada.ca/en/public-health/services/publications/healthy-living/canadian-immunization-guide-part-4-active-vaccines/page-9-human-papillomavirus-vaccine.html#a5_c. Accessed July 7, 2018.

[37] Badiga S , Chambers MM , Huh W , Eltoum IA , Piyathilake CJ . Expression of p16INK4A in Cervical Precancerous Lesions that are unlikely to be preventable by HPV Vaccines. Cancer. 2016;122:3615‐3623. 27479745 10.1002/cncr.30229 PMC5115942

[38] Mathews WC , Sitapati A , Caperna JC , Barber RE , Tugend A , Go U . Measurement characteristics of anal cytology, histopathology, and high‐resolution anoscopic visual impression in an anal dysplasia screening program. J Acquir Immune Defic Syndr. 2004;37:1610‐1615. 15577418 10.1097/00126334-200412150-00014

[39] Betancourt EM , Wahbah MM , Been LC , Chiao EY , Citron DR , Laucirica R . Anal cytology as a predictor of anal intraepithelial neoplasia in HIV‐positive men and women. Diagn Cytopathol. 2013;41:697‐702. 23288861 10.1002/dc.22941

[40] Panther LA , Wagner K , Proper J , et al. High resolution anoscopy findings for men who have sex with men: inaccuracy of anal cytology as a predictor of histologic high‐grade anal intraepithelial neoplasia and the impact of HIV serostatus. Clin Infect Dis. 2004;38:1490‐1492. 15156490 10.1086/383574

[41] Nahas CSR , da Silva Filho EV , Segurado AAC , et al. Screening anal dysplasia in HIV‐infected patients: is there an agreement between anal pap smear and high‐resolution anoscopy‐guided biopsy? Dis Colon Rectum. 2009;52:1854‐1860. 19966632 10.1007/DCR.0b013e3181b98f36

