# Supplementary material for: A rigorous exploration of anal HPV genotypes using a next‐generation sequencing (NGS) approach in HIV‐infected men who have sex with men at risk for developing anal cancer
Source: Cancer Med. 2019 Nov 25;9(2):807–15. doi: 10.1002/cam4.2720 (PMC6970045; doi:10.1002/cam4.2720)
Supplement: Supplementary file 1 [file CAM4-9-807-s001.pdf]

Supplementary Figure 1. An example of NGS HPV data shown as # and % reads of HPV genotypes detected in five anal specimens of HIV+ MSM

HPV genotype	Specimen 1		Specimen 2		Specimen 3		Specimen 4		Specimen 5	
	# of reads	% reads	# of reads	% reads	# of reads	% reads	# of reads	% reads	# of reads	% reads
HPV6**	5441	8.335	5	0.007	3	0.003	3	0.004	9	0.012
HPV7***	3	0.005	6	0.008	0	0	0	0	0	0
HPV11**	1	0.002	1484	1.967	1	0.001	3	0.004	13	0.017
HPV16*	29	0.044	36304	48.12	6	0.006	13472	15.85	75688	98.501
HPV18*	14	0.021	1	0.001	9	0.008	9	0.011	4	0.005
HPV26**	0	0	0	0	0	0	0	0	4	0.005
HPV30***	2	0.003	0	0	0	0	0	0	0	0
HPV31*	4	0.006	524	0.695	5	0.005	5	0.006	196	0.255
HPV32***	0	0	220	0.292	0	0	0	0	2	0.003
HPV33*	0	0	1	0.001	0	0	0	0	0	0
HPV35*	49528	75.868	7	0.009	21	0.02	69556	81.833	8	0.01
HPV39*	13	0.02	3	0.004	103344	95.973	1059	1.246	3	0.004
HPV44***	9	0.014	25	0.033	11	0.01	1	0.001	22	0.029
HPV45*	0	0	2	0.003	3	0.003	0	0	30	0.039
HPV51*	2	0.003	0	0	0	0	1	0.001	0	0
HPV52*	1	0.002	89	0.118	0	0	1	0.001	7	0.009
HPV53**	8	0.012	11	0.015	2	0.002	5	0.006	30	0.039
HPV56*	9548	14.626	0	0	2	0.002	3	0.004	0	0
HPV58*	2	0.003	0	0	0	0	2	0.002	1	0.001
HPV59*	1	0.002	0	0	0	0	0	0	4	0.005
HPV61**	0	0	0	0	0	0	0	0	14	0.018
HPV62**	4	0.006	18525	24.554	3	0.003	4	0.005	5	0.007
HPV66**	6	0.009	0	0	3161	2.936	5	0.006	1	0.001
HPV68*	0	0	0	0	0	0	0	0	0	0
HPV69**	0	0	5	0.007	2	0.002	1	0.001	3	0.004
HPV70**	1	0.002	15950	21.141	5	0.005	2	0.002	7	0.009
HPV72**	0	0	541	0.717	0	0	0	0	0	0
HPV73**	1	0.002	1	0.001	0	0	1	0.001	3	0.004
HPV74***	0	0	769	1.019	0	0	1	0.001	1	0.001
HPV81**	0	0	6	0.008	0	0	0	0	5	0.007
HPV82**	0	0	7	0.009	0	0	0	0	0	0
HPV83**	0	0	1	0.001	0	0	1	0.001	0	0
HPV84**	6	0.009	6	0.008	1	0.001	2	0.002	0	0
HPV85***	0	0	0	0	0	0	0	0	1	0.001
HPV114***	1	0.002	1	0.001	0	0	0	0	0	0
Other****	657	1.006	951	1.261	1101	1.022	860	1.012	779	1.014
Total	65282	100	75445	100	107680	100	84997	1000	76840	100

*Known HR-HPVs
**Known LR-HPVs
***Other HPVs documented in the PaVE database
****HPV sequences not documented in the PaVE database
