# Supplementary material for: A rigorous exploration of anal HPV genotypes using a next‐generation sequencing (NGS) approach in HIV‐infected men who have sex with men at risk for developing anal cancer
Source: Cancer Med. 2019 Nov 25;9(2):807–15. doi: 10.1002/cam4.2720 (PMC6970045; doi:10.1002/cam4.2720)
Supplement: Supplementary file 2 [file CAM4-9-807-s002.docx]

**Supplementary Table 1. The prevalence of HPV genotypes detected by % read cut points**

| **% reads cut points** | **13 known HR-HPVs** | **21 known LR-HPVs** | **12 Other HPVs documented in the PaVE database** | **HPV sequences not documented in the PaVE database** |
| --- | --- | --- | --- | --- |
| >0% | 16 (100%), 18 (95%), 31 (70%), 33 (22%), 35 (72%), 39 (79%), 45 (80%), 51 (27%), 52 (81%), 56 (42%), 58 (70%), 59 (62%), 68 (17%) | 6 (95%), 11 (85%), 26 (60%), 40 (32%), 42 (51%), 53 (88%), 54 (22%), 61 (54%), 62 (96%), 66 (86%), 67 (23%), 69 (49%), 70 (78%), 71 (2%), 72 (47%), 73 (42%), 81 (79%), 82 (33%), 83(63%), 84 (31%), 89 (30%) | 102 (2%), 114 (49%), 27 (1%), 30 (2%), 32 (23%), 43 (19%), 44 (80%), 7 (7%), 74 (64%), 85 (32%), 86 (2%), 97(1%) | 100% |
| >5% | 16 (25%), 18 (11%), 31 (9%), 35 (10%), 39 (6%), 45 (9%), 51 (2%), 52 (10%), 56 (2%), 58 (6%), 59 (7%), 68 (1%) | 6 (22%), 11 (10%), 26 (4%), 40 (2%), 42 (4%), 53 (14%), 61 (5%), 62 (16%), 66 (9%), 67 (1%), 69 (4%), 70 (7%), 72 (4%), 73 (1%), 81 (7%), 82 (4%), 83 (4%), 89 (1%) | 114 (2%), 32 (1%), 43 (1%), 74 (4 %) 44 (9%), 85 (1%) | 25% |
| >10% | 16 (20%), 18 (9%), 31 (6%), 35 (9%), 39 (5%), 45 (6%), 51(1%), 52(6%), 56 (2%), 58 (5%), 59 (6%), 68 (1%) | 6 (19%), 11 (9%), 26 (2%), 40 (2%), 42 (2%), 53 (11%), 61 (4%), 62 (14%), 66 (9%), 67 (1%), 69 (4%), 70 (7%), 72 (2%), 81 (5%), 82 (2%), 83 (4%), 89 (1%) | 114 (1%), 32 (1%), 44 (5%), 85 (1%) | 22% |
| >20% | 16 (16%), 18 (4%), 31 (4%), 35 (7%), 39 (5%), 45 (5%), 52 (1%), 56 (1%), 58 (5%), 59 (1%), 68 (1%) | 6 (17%), 11 (9%), 26 (2%), 40 (2%), 53 (9%), 61 (1%), 62(10%), 66 (7%), 67 (1%), 69 (2%), 70 (4%), 81(4%), 82 (1%), 83 (2%) | 114 (1%), 32 (1%), 44 (4%), 85 (1%) | 11% |
| >30% | 16 (15%), 18 (4%), 31 (4%), 35 (6%), 39 (5%), 45 (4%), 52 (1%), 56 (1%), 58 (5%), 59 (1%) | 6 (15%), 11 (7%), 26 (2%), 40 (2%), 53 (9%), 62 (6%), 66 (6%), 67 (1%), 69 (1%), 70 (1%), 81 (1%), 82 (1%), 83 (1%) | 114 (1%), 44 (4%) | 6% |
